# Long noncoding RNA HOST2, working as a competitive endogenous RNA, promotes STAT3-mediated cell proliferation and migration via decoying of let-7b in triple-negative breast cancer

**DOI:** 10.1186/s13046-020-01561-7

**Published:** 2020-04-05

**Authors:** Kaiyao Hua, Xiaochong Deng, Jiashu Hu, Changle Ji, Yunhe Yu, Jiayi Li, Xuehui Wang, Lin Fang

**Affiliations:** 1 Department of Breast and Thyroid Surgery, Shanghai Tenth People’s Hospital, School of Medicine, Tongji University, Shanghai, 200072 China; 2 Nanjing Medical University, Nanjing, 210029 China; 3 Medical College of Soochow University, Suzhou, 215006 China

**Keywords:** HOST2, TNBC, Let-7b, STAT3, ceRNA

## Abstract

**Background:**

Human ovarian cancer specific transcript 2 (HOST2) is a long non-coding RNA (lncRNA) reported to be specifically high expressed in human ovarian cancer. However, the mechanism that how HOST2 regulates triple negative breast cancer (TNBC) need to be explored.

**Methods:**

In this study, expression of HOST2 was determined in 40 TNBC patients and matched non-cancerous tissues by qRT-PCR and in situ hybridization (ISH) assay. The biological functions of HOST2 was measured by losing features. The effect of HOST2 on viability, proliferation and migration was evaluated by MTT, colony formation assay, EDU analysis, transwell invasion assay and nude mouse xenograft model. Fluorescence in situ hybridization (FISH), Luciferase report assay, RNA immunoprecipitation (RIP) assay and Western blot were fulfilled to measure molecular mechanisms.

**Results:**

The results showed that HOST2 was up-regulated in BC tissues and cell lines. Clinical outcome analysis demonstrated that high expression of HOST2 was associated with poor prognosis of TNBC patients. Functional experiments illustrated that knockdown of HOST2 significantly suppressed TNBC cell proliferation and migration. Western blot assays, qRT-PCR assays, RIP assays and luciferase reporter assays revealed that HOST2 regulated STAT3 via crosstalk with let-7b. Depression of HOST2 suppressed STAT3-mediated proliferation and migration in TNBC cells. HOST2 could function as a decoy of let-7b to depress expression of STAT3.

**Conclusions:**

HOST2 could function as a oncogene and promoted STAT3-mediated proliferation and migration through acting as a competing endogenous RNA, which might act as a potential biomarker for TNBC patients.

## Background

The incidence of breast cancer (BC) is rising rapidly with more than 1 million new cases diagnosed annually [1]. Based on molecular characterization of estrogen receptor (ER), progesterone receptor (PR), ki-67, and HER-2, Breast cancer can be divided into different types including Lumina A, Lumina B, HER-2 overexpression, basal-like tumours, and ‘normal-like’ breast tumours. TNBC is defined as a tumour that is negative for ER, PR, and HER-2 [2]. The main systemic treatment for TNBC patients currently still is surgical resection and chemotherapy because of lacking meaningful hormonal biomarker [3]. The metastasis and recurrence rates of TNBC patients are significantly higher compared with other breast cancer subtypes [4, 5]. So, exploring more effective target for early diagnosis and treatment of TNBC is essential.

LncRNAs, a new kind of non-coding RNA, are longer than 200 nucleotides in length with no or limited protein-coding potential [6]. LncRNAs have been proved to play critical function in tumorigenesis through transcriptional levels to post-transcriptional levels [7, 8]. lncRNAs are able to regulate the proliferation, apoptosis, migration and invasion of human cancer cells [9, 10]. For instance, lncRNA-DANCR regulates proliferation and migration by epigenetically silencing FBP1 in tumourigenesis of cholangiocarcinoma [11]. LncRNA-SNHG6 enhances cell proliferation, migration and invasion by regulating miR-26a-5p/MAPK6 in breast cancer [12]. LncRNAs, so far, have been proved to be a keynote in breast cancer research [13–15]. Therefore, to better understand the pathogenesis of TNBC, elucidating the mechanism of lncRNAs turns to be essential.

MicroRNAs usually acted as vital regulators of their target genes by binding to 3′-untranslated regions (3′-UTRs) [16]. Recently, competing endogenous RNAs (ceRNAs) hypothesis was discovered that lncRNAs could regulate the expression of certain cancer related genes by lncRNA-miRNA or lncRNA-mRNA interaction [17]. Aberrant expression of miRNAs is reported in various types of cancers including TNBC. For example, miR-21, miR-221/222, miR-373 are reported to be up-regulated while miR-145, miR-199a-5p, miR-200 family are down-regulated in TNBC [18]. In the cytoplasm, lncRNAs can work as ceRNAs by binding to certain miRNAs to modulate the depression of miRNAs targets [19–21].

HOST2 is a novel lncRNA with a length of 2.9 kb, without an obvious open reading frame (ORF) [22]. In 2015, HOST2 was firstly reported in human ovarian cancer which is highly expressed, and function as an oncogene in epithelial ovarian cancer cells by binding to miRNA let-7b [23]. In hepatocellular carcinoma [24, 25], cervical cancer [26], gastric cancer [27] and glioma [28], HOST2 was also up-regulated and played a oncogene role in tumourigenesis and development. However, there is still lack of exact mechanisms about how HOST2 experts its functions in TNBC.

In our study, firstly, the expression of HOST2 was detected in TNBC tissues and their paired noncancerous tissues. By analyzing HOST2 through clinical data, we concluded that high expression of HOST2 was significantly associated with later pathological staging and poor survival of TNBC patients. Further functional studies indicated that knockdown of HOST2 inhibited TNBC cell growth and migration in vitro and tumour growth in vivo. More importantly, we found that HOST2 exert its oncogenic role in TNBC cells via sponging tumour suppressor let-7b and miR-1266 to upregulate STAT3 signaling pathway. These findings provided a new insights into the treatment and diagnosis of TNBC.

## Materials and methods

### Clinical samples

In our study, 40 TNBC patients cancer tissues and their adjacent noncancerous tissues were collected. All the specimens were collected from the Department of Breast and Thyroid Surgery of Shanghai Tenth People’s Hospital, China. The specimens were snap-frozen in liquid nitrogen and these patients had not received any anti-cancer treatment bofore surgery. Our study protocols were approved by Institutional Ethics Committees of Shanghai Tenth People’s Hospital.

### Cell culture and transfection

The human breast cancer cell lines MDA-MB-231, MDA-MB-468, HCC1937, MCF-7 and SKBR3 were purchased from the Chinese Academy of Sciences. These cells were cultured in Dulbecco’s modified Eagle’s medium (DMEM; Gibco; Thermo Fisher Scientific, Inc.) supplemented with 10% fetal bovine serum (FBS; Gibco; Thermo Fisher Scientific, Inc.), penicillin (100 U/ml) and streptomycin (100 μg/ml) (PS; Enpromise, Hangzhou, China). Human normal breast cell line MCF-10A was bought from Shanghai Zhongqiao Xinzhou Biotechnology Co., Ltd. MCF-10A cells were cultured in Mammary Epithelial Cell Medium (MEpiCm, ScienCell, Research Laboratories, Inc.). All these cells were incubated at 37 °C supplemented with 5% CO_2_.

In vitro experiment, Let-7b mimics (let-7b), miR-1266 mimics (miR-1266) and negative control (miR-NC) were synthesized by RiboBio (Guangzhou, China). let-7b sense: 5′-CUAUACAACCUACUGCCUUCCC-3′, antisence: 5′-GGGAAGGCAGUAGGUUGUAUAG-3′; miR-1266 sense: 5′-CCUCAGGGCUGUAGAACAGGGCU-3′, antisence: 5′-AGCCCUGUUCUACAGCCCUGAGG-3′; miR-NC sense: 5′-UUCUCCGAACGUGUCACGUTT-3′, antisence 5′-ACGUGACACGUUCGGAGAATT-3′. HOST2 siRNA (si-HOST2) and negative controls (siRNA-NC) were synthesized by Sangon Biotech (Shanghai, China). si-HOST2 sense: 5′-GACUAAACAAGGUCUUAAUTT-3′, antisense: 5′-AUUAAGACCUUGUUUAGUCTT-3′. siRNA-NC sense: 5′-UUCUCCGAACGUGUCACGUTT-3′, antisense: 5′-ACGUGACACGUUCGGAGAATT-3′. In vivo experiment, HOST2 shRNA (sh-HOST2) and NC (sh-NC) were also synthesized by RiboBio (Guangzhou, China), MDA-MB-231 cells were infected with lentiviruses. All siRNAs, miRNAs and and negative control were transfected into cells using Lipofectamine 2000.

### RNA extraction and quantitative real-time PCR (qRT-PCR)

Total RNA was extracted from MDA-MB-231, MDA-MB-468, HCC1937, MCF-7 and SKBR3 and MCF-10A cells by TRIzol® reagent (Invitrogen; Thermo Fisher Scientific, Inc.) according to the manufacturer’s protocol. cDNA was generated via reverse transcription using the PrimeScript RT-PCR kit in accordance with the manufacturer’s instructions (Takara Bio, Inc.). Subsequently, qPCR was performed on a 7900HT Fast RT-PCR instrument (Applied Biosystems; Thermo Fisher Scientific, Inc.). The primer sequences were synthesized by RiboBio (Guangzhou, China). HOST2, sense: 5′-GGACAGGTCCCTTGTTTCAA-3′, antisence: 5′- CTGGTCTTTCCTTGCCTCTG-3′; GAPDH, sense:5′- CCACTCCTCCACCTTTGAC − 3′, antisence: 5′-ACCCTGTTGCTGTAGCCA − 3′. The amplification protocol was as follows: Initial denaturation for 3 min at 95 °C, followed by 40 cycles of denaturation at 95 °C for 3 s, annealing at 65 °C for 30 s and elongation at 72 °C for 20 s. Expression of mRNAs or miRNAs was assessed by handling threshold cycle (CT) values. The relative expression levels were counted by a 2^−ΔΔCt^ method.

### Cell proliferation assays

For MTT assay, the transfected cells were seeded into 96-well plates at a density of 500 cells/well. Cell viability was estimated using an MTT assay kit (Sangon Biotech Co., Ltd.) at 24, 48, 72, and 96 h, according to the manufacturer’s instructions. After 4 h incubation in MTT reagent at 37 °C and 5% CO_2_, the medium was replaced with 150 μl dimethyl sulfoxide at room temperature (DMSO; Sangon Biotech Co., Ltd.). The absorbance of each sample was measured at 490 nm using a microplate spectrophotometer (BioTek Instruments, Inc.), after 10 min of agitation on a shaking table.

For colony formation assay, the treated cells were then harvested and plated into a six-well plate at a density of 500 cells/well. The plates were incubated at 37 °C and 5% CO_2_ for 7 to 10 days, with the medium changed every three days. When the colonies were visible, the medium was removed and the plates were washed three times with phosphate buffered saline (PBS) and allowed to dry. The dried colonies were fixed using 95% ethanol for 15 min at room temperature, then dried and stained with 0.1% crystal violet solution for 15 min at room temperature. Finally, the colonies were washed with water three times, dried and immediately imaged. Colonies were counted using a light microscope (magnification, × 20).

For EDU analysis, the transfected cells (5 × 10^3^ cells/well) were firstly transferred into 96-well plates. 5-Ethynyl-2-deoxyuridine (EDU) labeling/detection kit (Ribobio, Guangzhou, China) was used according to the protocol. We took representative pictures with a microscope and analyzed the pictures using Image J.

### Transwell assays

For transwell assay, the experiment was conducted by a 24-well insert with its upper chambers Matrigel-coated (BD Bioscience, New Jersey, USA). Cells were placed in the upper chamber with serum-free medium. Then Medium was added with 10% FBS to the lower chamber. 24 h later, the transwells were fixed with 4% paraformaldehyde staining with 0.05% crystal violet. Representative pictures were taken with a microscope and stained cells were counted in five random fields.

### Dual-luciferase reporter assay

293 T cells (Shanghai Institute of Biochemistry and Cell Biology) were seeded in 48-well plates and incubated at 37 °C in a cell incubator supplemented with 5% CO_2_. When cell confluency reached 80%, DMEM medium was replaced with medium without FBS or PS (250 μl/well). Wild and mutant reporter plasmids of HOST2 and STAT3 which containing a wild or mutant let-7b or miR-1266 binding sites were individually purchased from Integrated Biotech Solutions. 293 T cells were co-transfected with HOST2 and STAT3 mutant and wild type reporter plasmids, and together with let-7b mimics/miR-1266 mimics or mimic control vector using Lipofectamine® 2000 reagent. 48 h later, firefly and Renilla luciferase activities were measured using a Dual-Luciferase® Reporter Assay kit (Promega, Madison, WI, USA). Firefly luciferase activity was normalized to the Renilla control, and the ratio of firefly/Renilla activity was recorded.

### Western blot analysis

We extracted proteins by using RIPA lysis buffer (Beyotime, Jiangsu, China). Their concentrations were detected by using a BCA protein assay kit (Beyotime). Protein samples were separated by electrophoresis on a 8% or 10% polyacrylamide SDS gel (Beyotime Institute of Biotechnology) and transferred onto 0.45 μm nitrocellulose membranes (Beyotime Institute of Biotechnology). The the membranes were washed with PBS adding 0.1% tween20 (PBST) and incubated with 5% skimmed milk for 1 h. Membranes were incubated with primary antibodies in antibody diluent (Beyotime Institute of Biotechnology) overnight at 4 °C. The following primary antibodies were used: STAT3 (1:1000; Bioworld Technology, China), and GAPDH (1,1000, Bioworld Technology, China). The next day, we washed and incubated the membranes in secondary antibodies for 1 h. Immunoreactive protein bands were scaned by a Odyssey scanning system (Li-Cor, Lincoln, NE, USA).

### In situ hybridization (ISH), fluorescence in situ hybridization (FISH), and Immunohistochemical (IHC) staining

For ISH, Biotinylated probes were used to measure HOST2 expression in TNBC tissue. Biotin-binding probes used in this experiment were purchased from RiboBio (Guangzhou, China). In situ hybridization assay was performed on fresh TNBC tissue slices. The treated slices were incubated with anti-HOST2 oligodeoxy-nucleotide probes (RiboBio, Guangzhou, China) with hybridization solution containing 1% blocking solution in humid chamber at 37 °C overnight. The next day, slices were subsequently washed at 42 °C with 0.1% Tween-20 in 4× sodium citrate buffer (SSC), 2× SSC, and 1× SSC. The sections were then stained with hematoxylin and dehydrated in graded alcohols and xylene.

For FISH assay, Ribo™ Fluorescent In Situ Hybridization Kit (Ribo, China) was used. Ribo™ lncRNA FISH Probe Mix was synthesized in our experiment. BC cells were grown on the coverslips and cells were treated by glycine, following by acetylation reagent. The cells were incubated in prehybridization solution, then hybridization solution containing probe (300 ng/mL) was added and incubated overnight at 42 °C. After washing in PBST, we used 4′,6-Diamidino-2-Phenylindole (DAPI) to sign the nucleus. Fluorescence microscope was used to capture the images of cells.

For IHC assay, We firstly cut the specimens and placed them on slides. Xylenes was used to deparaffinize the sections and were rehydrated by graded ethanol washes. Then we placed the slides with 90 °C antigen retrieval buffer for 10 min and cooling at 25 °C. The slides were blocked in 3% H_2_O_2_ and 5% BSA at 25 °C. Then the slides were incubated overnight in primary antibody. After then, we incubated the slides in Secondary antibody. Finally, representative pictures were taken with a microscope of each slice.

### Nuclear fractionation

Nuclear fractionation was performed by using a PARIS™ Kit (Ambion, Austin, TX). 1 × 10^7^ cells (MDA-MB-231 or MDA-MB-468) were collected and then resuspended in the cell fraction buffer. The cells were incubated on ice for 10 min. According to the manufacturer’ s instructions. Supernatant and nuclear pellets were separated with cell disruption buffer for RNA extraction.

### RIP assay

RIP assay was performed using Magna RNA-binding protein immunoprecipitation kit (Millipore, USA). RNA concentration was detected by spectrophotometer (Thermo Scientific, USA), RNA quality was detected by bio-analyzer (Agilent, USA). The Input in this experiment is total cell lysate by TRIzol. qRT-PCR was conducted to assess the purified RNAs so as to demonstrate the targets.

### Mice model experiment

Athymic nude mice were ordered from Shanghai laboratory animal center. Lentiviruses and puromycin were used in the construction of HOST2 (sh-HOST2) or NC (sh-NC) MDA-MB-231 cells. Cells (5 × 10^6^ per mice) were overexpressed by HOST2 (sh-HOST2) or NC (sh-NC) and digested into cells suspension. Then we injected the cells suspension into the second mammary fat of the mice (*n* = 4, each group). Tumor size was measured every week. The tumors were collected 5 weeks after treating.

### Statistical analysis

The significance of differences between groups was assessed by Student’s t-test, one-way ANOVAs or χ^2^ test. Survival curves were estimated by the Kaplan-Meier method. The log-rank test was used to determine the statistical differences between survival curves. All statistical analyses were performed using GraphPad Prism V6.0 (GraphPad Software, Inc., La Jolla, CA, USA) and SPSS 20.0 (IBM, SPSS, Chicago, IL, USA). All experiments were independently repeated three times. Significantly differences were considered for *P*-values < 0.05.

## Results

### HOST2, a cytoplasmic lncRNA, was up-regulated in TNBC tissues and cell lines and was correlated with poor prognosis in TNBC patients

**Fig. 1:**
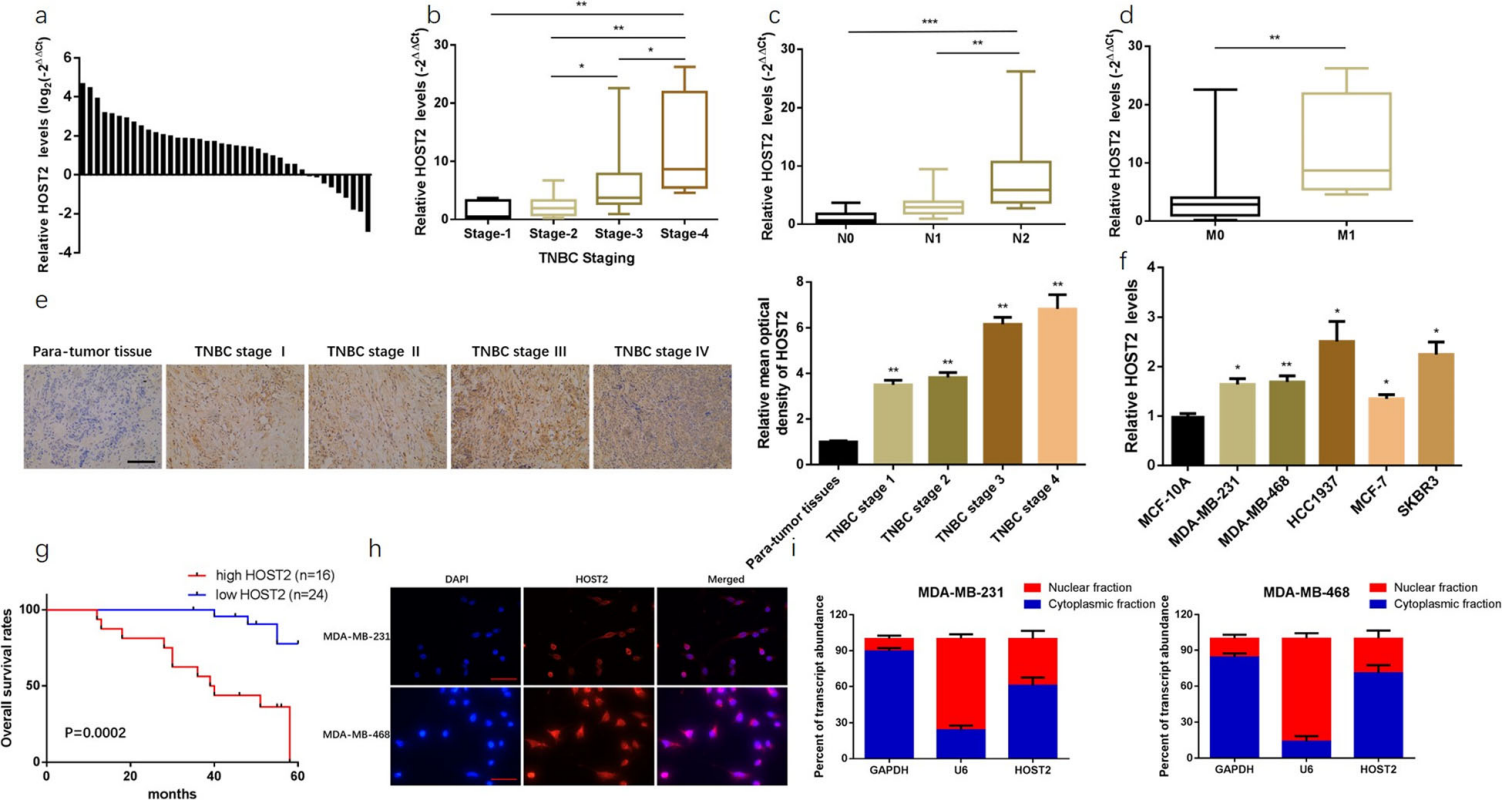
HOST2 was upregulated in TNBC tissues and associated with poor survival. **a** The expression of HOST2 in collected 40 cases of TNBC tissues and matched non-cancerous breast tissues indicated by qRT-PCR analysis. **b-d** The expression of HOST2 in TNBC patients with advanced stage, lymph node metastasis and distant metastasis. **e** The expression of HOST2 was detected in different TNM stage by In situ hybridization analysis comparing with para-tumour tissue group respectively (200×). **f** The expression of HOST2 in breast cancer cell lines contrasted to MCF-10A group, data was normalized to MCF-10A group. **g** The correlation of HOST2 high expression or low expression with overall survival in TNBC patients determined by Kaplan-Meier analysis. **h** FISH assay revealed that HOST2 was mostly stained in TNBC cells’ cytoplasm (200×). **i** Subcellular fractionation demonstrated that HOST2 was enriched in the cytoplasm of TNBC cells. ^*^P < 0.05, ^**^P < 0.01, ^***^P < 0.001. All experiments were repeated three times

The expression of HOST2 in 40 paired TNBC tissues and non-cancerous breast tissues were determined by qRT-PCR. The results showed that HOST2 was significantly elevated in most TNBC tissues (31/40, 77.5%) compared with para-tumour tissues (Fig. 1a). Moreover, the expression of HOST2 were significantly increased in three TNBC cell lines (MDA-MB-231, MDA-MB-468 and HCC1937) compared to MCF-10A (Fig. 1f). The similar trend was also showed in MCF-7 (Lumina A type) and SKBR3 (Her-2 overexpression type) cells (Fig. 1f).

To further investigated the clinicopathological and prognostic significance of HOST2 in TNBC patients, clinical data of the 40 TNBC patients was collected and analyzed together with HOST2 levels. As the outcomes displayed in Fig. 1b-d, the expression of HOST2 was notably higher in TNBC patients with advanced stage (Fig. 1b), lymph node metastasis (Fig. 1c) and distant metastasis (Fig. 1d). The identification of the demarcation point to distinguish high or low expression of HOST2 was obtained by drawing ROC curve. The sensitivity, specificity, and Youden index was calculated. The demarcation point of HOST2 expression locates in the maximum of Youden index, and this value is taken as the demarcation point to distinguish high or low expression of HOST2 (Figure S1). Kaplan-Meier analysis was used to evaluate the correlation between HOST2 expression and the OS (Overall Survival) of TNBC patients. The results suggested that the TNBC patients with HOST2 high expression had a poorer survival compared to those with HOST2 low expression (Fig. 1g).

In addition, ISH assay was applied to evaluate the expression of HOST2 in TNBC tissues. As shown in Fig. 1e, HOST2 was more stained in TNBC advanced stage (Fig. 1e). Then the localization of HOST2 in cytoplasm or nucleus of TNBC cells was identified with FISH assay and subcellular fractionation. FISH analysis revealed that HOST2 was mostly stained in TNBC cells’ cytoplasm (Fig. 1h). Subcellular fractionation also demonstrated quantitatively that HOST2 was enriched in the cytoplasm of TNBC cells (Fig. 1i).

### HOST2 promoted proliferation and invasion of TNBC cells

**Fig. 2:**
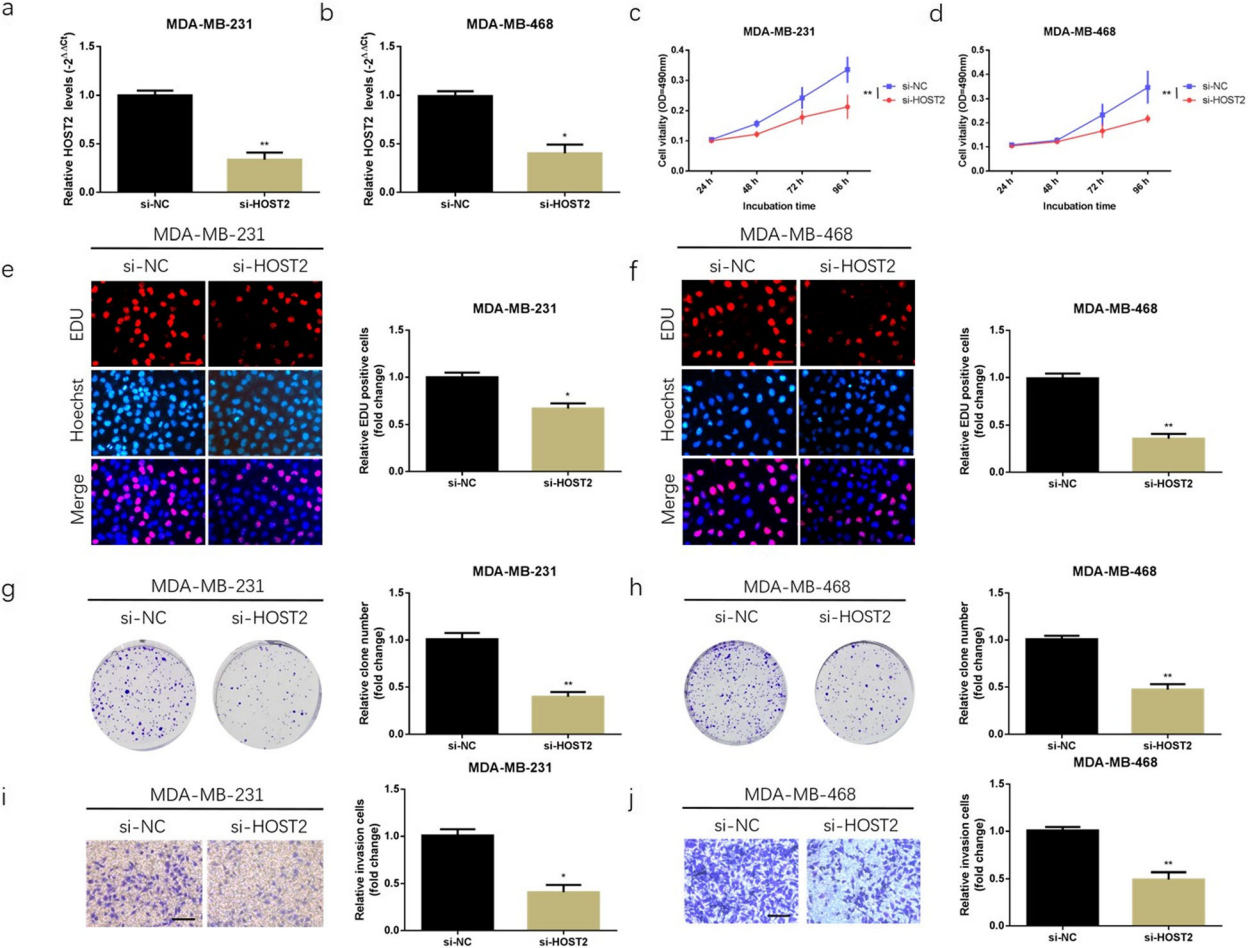
HOST2 promoted proliferation and invasion of TNBC cells in vitro. **a, b** qRT-PCR was conducted to verify the relative expression of HOST2 in MDA-MB-231 and MDA-MB-468 cells transfected with HOST2-siRNA (si-HOST2). **c, d** MTT assay was performed to determine the cell viability in MDA-MB-231 and MDA-MB-468 cells after knockdown of HOST2. **e, f** Representative results of the EDU assay of MDA-MB-231 and MDA-MB-468 cells after knockdown of HOST2. **g, h** Effects of HOST2 knockdown on cell colony formation ability in MDA-MB-231 and MDA-MB-468 cells. **i, j** Representative results of transwell invasion assay of MDA-MB-231 and MDA-MB-468 cells after knockdown of HOST2. ^*^P < 0.05, ^**^P < 0.01. All experiments were repeated three times

MDA-MB-231 and MDA-MB-468 cells were transfected with HOST2-siRNA (si-HOST2). qRT-PCR analysis was used to confirm that HOST2 expression was effectively modulated in MDA-MB-231 and MDA-MB-468 cells (Fig. 2a, b). Knockdown of HOST2 reduced the colony number and lowered MTT activity compared with the NC group (Fig. 2c, d, g, h). Similarly, EDU assay also demonstrated that knockdown of HOST2 had a significant repression on TNBC cell proliferation (Fig. 2e, f). To observe the effects of HOST2 on TNBC cell migration, a transwell invasion assay was conducted. The results showed that knockdown of HOST2 declined cell invasive ability in MDA-MB-231 and MDA-MB-468 cell lines (Fig. 2i, j).

### HOST2 acted as a sponge of let-7b and miR-1266 in TNBC cells

**Fig. 3:**
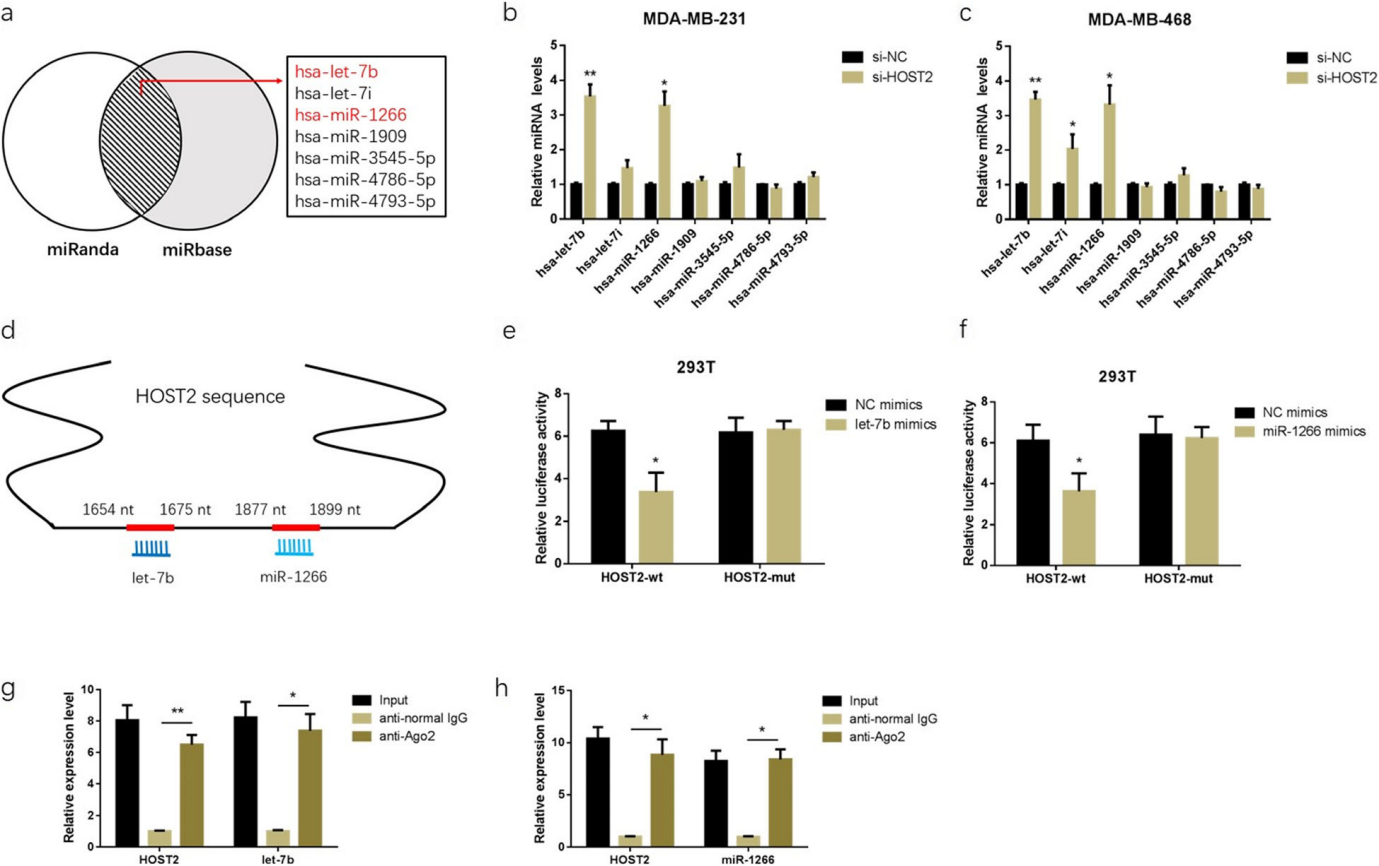
HOST2 serves as a let-7b and miR-1266 sponge in TNBC cells. **a** Seven potential target miRNAs of HOST2 were predicted by miRanda and miRbase. **b, c** The expression of seven potential target miRNAs in MDA-MB-231 and MDA-MB-468 cells after knockdowns of HOST2. **d** Schematic model showed the putative binding sites of let-7b and miR-1266 associated with HOST2. **e, f** Luciferase reporter assay in HEK293 T cells co-transfected with let-7b/miR-1266 mimics, psiCHECK-2-wild type HOST2 (HOST2-wt) or psiCHECk-2-mutant type HOST2 (HOST2-mut) plasmids. **g, h** RIP binding assay was performed using input from cell lysate, normal mouse IgG or anti-Ago2. Relative expression levels of HOST2 and let-7b/miR-1266 were determined by qRT-PCR. ^*^P < 0.05, ^**^P < 0.01. All experiments were repeated three times

Due to lncRNAs usually associated with miRNA sponging in the cytoplasm [29, 30], we further explored whether HOST2 could bind to miRNAs in TNBC. Seven potential target miRNAs (let-7b, let-7i, miR-1266, miR-1909, miR-3545-5p, miR-4786-5p, miR-4793-5p) of HOST2 were predicted by miRanda (http://www.miranda.org) and miRbase (http://www.mirbase.org), and these miRNAs were selected as miRNAs candidate for our following experiments (Fig. 3a). The positions of putative binding sites in HOST2 were shown in Fig. 3d. mRNA expression difference of the seven miRNAs was detected by qRT-PCR after knockdown HOST2 expression. Among the seven miRNAs candidate, let-7b and miR-1266 were abundantly increased in both MDA-MB-231 and MDA-MB-468 cells (Fig. 3b, c). Here, luciferase assay was conducted to detect the specific binding sites between HOST2 and let-7b/miR-1266. Compare to NC mimics, transfection of let-7b/miR-1266 mimics and HOST2-wt led to a prominent decrease of fluorescence. When the theoretical binding sites HOST2 might provide for let-7b/miR-1266 were mutated (cotranfection of let-7b/miR-1266 mimics and HOST2-mut), the fluorescence was restrengthened (Fig. 3e, f). Ago2, an important component of RNA induced silencing complex (RISC), generally interacts with RNAs [31, 32]. Then a RIP binding assay was conducted by using anti-Ago2 antibody in 293 T cell line, the results showed that the level of HOST2 and let-7b/miR-1266 was higher in anti-Ago2 group than that in anti-normal IgG group (Fig. 3g, h), indicating that HOST2 and let-7b/miR-1266 were in the same RISC. Overall, the result confirmed that HOST2 acted as a sponge of let-7b and miR-1266 in TNBC cells.

### Let-7b, rather than miR-1266, inhibited TNBC cell proliferation and invasion by binding to STAT3

**Fig. 4:**
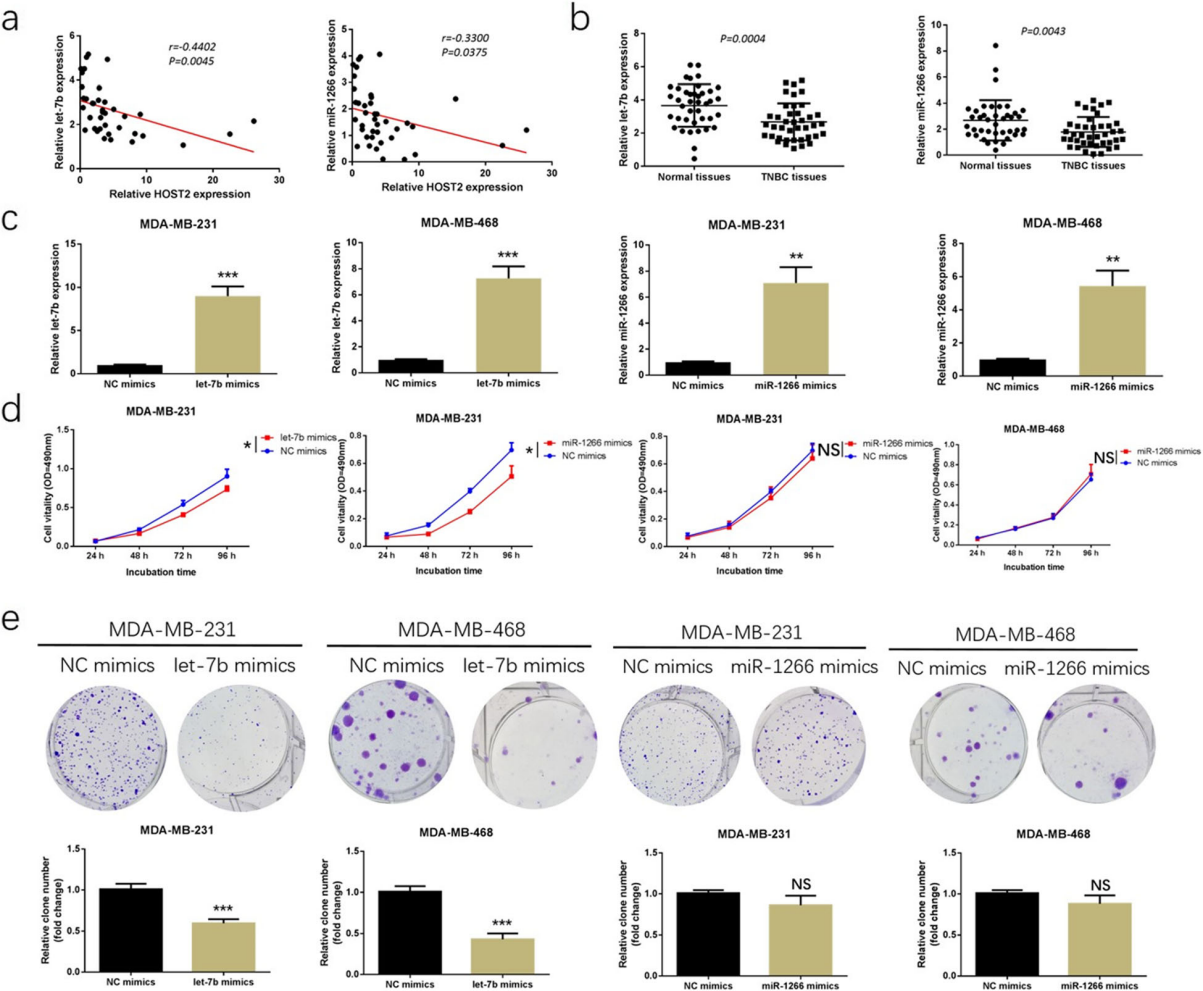
Let-7b, rather than miR-1266, inhibited TNBC cell proliferation. **a** Pearson correlation analysis of the correlation of HOST2 expression with let-7b and miR-1266 expression in the 40 TNBC samples. **b** Let-7b and miR-1266 expression in the 40 collected TNBC tissues compared to matched non-cancerous breast tissues. **c** Let-7b and miR-1266 expression was effectively modulated in MDA-MB-231 and MDA-MB-468 cells after let-7b/miR-1266 mimics transfection. **d** Effects of let-7b and miR-1266 overexpression on cell proliferation in MDA-MB-231 and MDA-MB-468 cells detected by MTT assay. **e** Effects of let-7b and miR-1266 expression on cell colony formation ability in MDA-MB-231 and MDA-MB-468 cells. ^*^P < 0.05, ^**^P < 0.01, ^***^P < 0.001

**Fig. 5:**
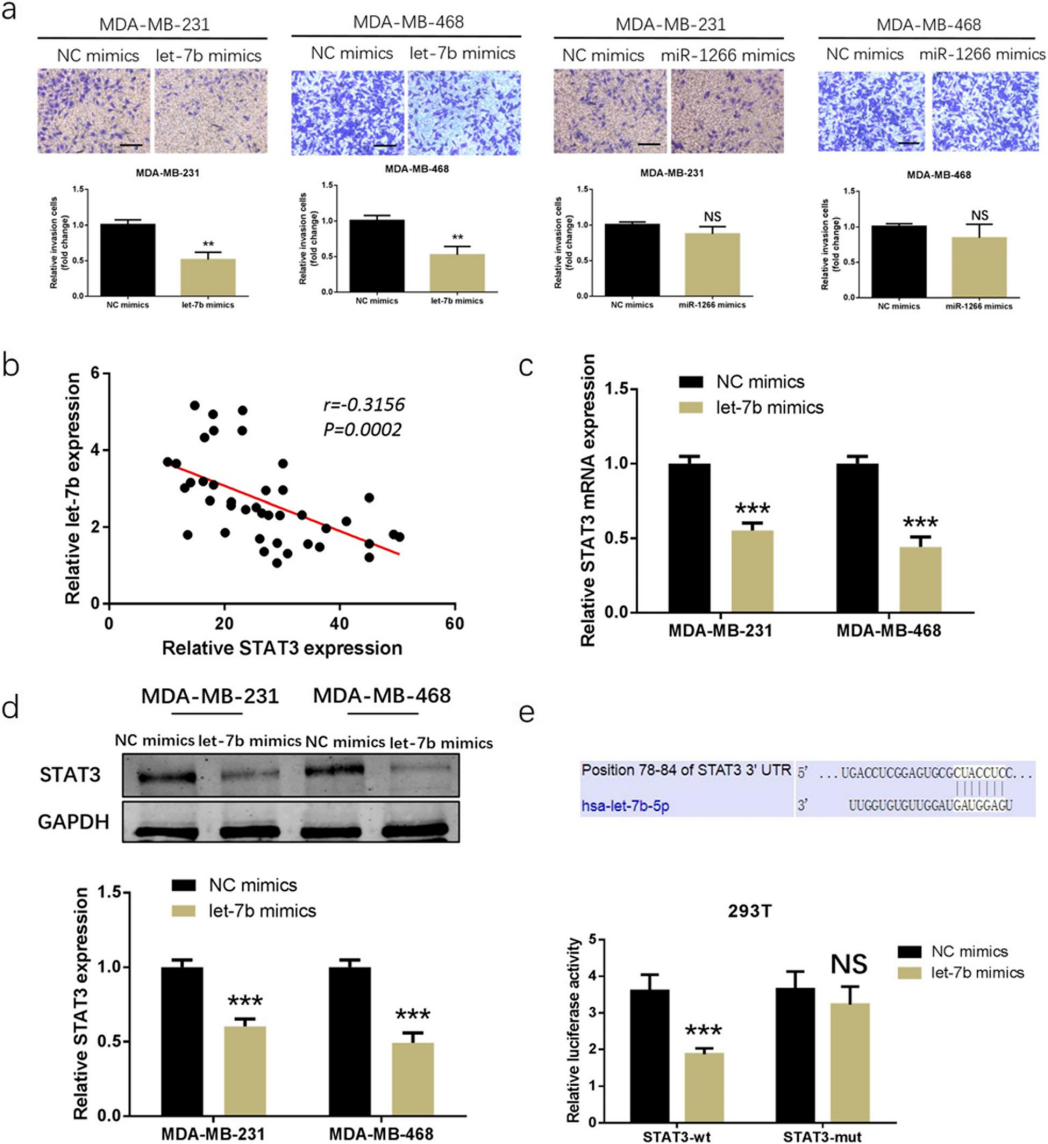
Let-7b, rather than miR-1266, inhibited TNBC cell invasion and STAT3 is a direct target of let-7b. **a** Effects of let-7b and miR-1266 overexpression on cell invasion in MDA-MB-231 and MDA-MB-468 cells detected by transwell invasion assay. **b** Pearson correlation analysis of the correlation of STAT3 expression with let-7b expression in the 40 TNBC samples. **c, d** The expression of STAT3 mRNA and protein after let-7b overexpression in TNBC cells detected by qRT-PCR and Western blot analysis. **e** The luciferase activity of STAT3-wt or STAT3-mut after co-transfection with let-7b mimics and STAT3-wt or STAT3-mut reporter vector. ^**^P < 0.01, ^***^P < 0.001

The particular effect and mechanism of let-7b and miR-1266 in TNBC was unclear. Firstly, correlations between HOST2 and let-7b/miR-1266 were observed by Pearson correlation analysis in the collected 40 cases of TNBC tissues. As shown in Fig. 4a, a negative correlation was found between HOST2 and let-7b/miR-1266. Secondly, the expression of let-7b and miR-1266 in TNBC tissues and matched non-cancerous breast tissues were determined by qRT-PCR. Let-7b and miR-1266 were lower expressed in the 40 TNBC tissues compared to matched non-cancerous breast tissues (Fig. 4b). Thirdly, let-7b and miR-1266 mimics were transfected into MDA-MB-231 and MDA-MB-468 cells and confirmed by qRT-PCR (Fig. 4c). MTT assay and colony formation assay indicated that overexpression of let-7b in MDA-MB-231 and MDA-MB-468 cells promoted cell proliferation, while overexpression of miR-1266 had no obvious effect on cell proliferation (Fig. 4d, e). Moreover, as shown in Fig. 5a, through transwell invasion assays, the number of MDA-MB-231 and MDA-MB-468 cells penetrating the membrane significantly decreased at 24 h in let-7b group compared to the NC group. However, there was no obvious difference between miR-1266 and NC group. These results suggested that let-7b has more effects in TNBC. The potential target of let-7b need to be found. Combined the prediction scores of starBase3.0 (http://starbase.sysu.edu.cn) and targetscan database (http://www.targetscan.org), STAT3, an oncogene in TNBC, could be a potential target of let-7b. After that, the correlation between the expression of let-7b and STAT3 was investigated in the collected TNBC tissues. We found that there was a markedly inverse correlation between let-7b and STAT3 expression (Fig. 5b), further indicating that STAT3 is a potential target of let-7b. Furthermore, let-7b could negatively regulate STAT3 mRNA and protein expression (Fig. 5c, d). More importantly, luciferase reporter assay was applied through psi-Check2 vector holding wild-type or mutant 3′-UTR of STAT3. In STAT3-wt group, luciferase activities decreased after adding let-7b mimics in 293 T cells, while no prominent differences were found for mutant 3′-UTR of STAT3 (Fig. 5e), suggesting that let-7b specifically binds to the 3′-UTR of STAT3 mRNA.

### HOST2 knockdown-induced tumour suppressing effects in TNBC cells could be reversed by inhibiting let-7b expression

**Fig. 6:**
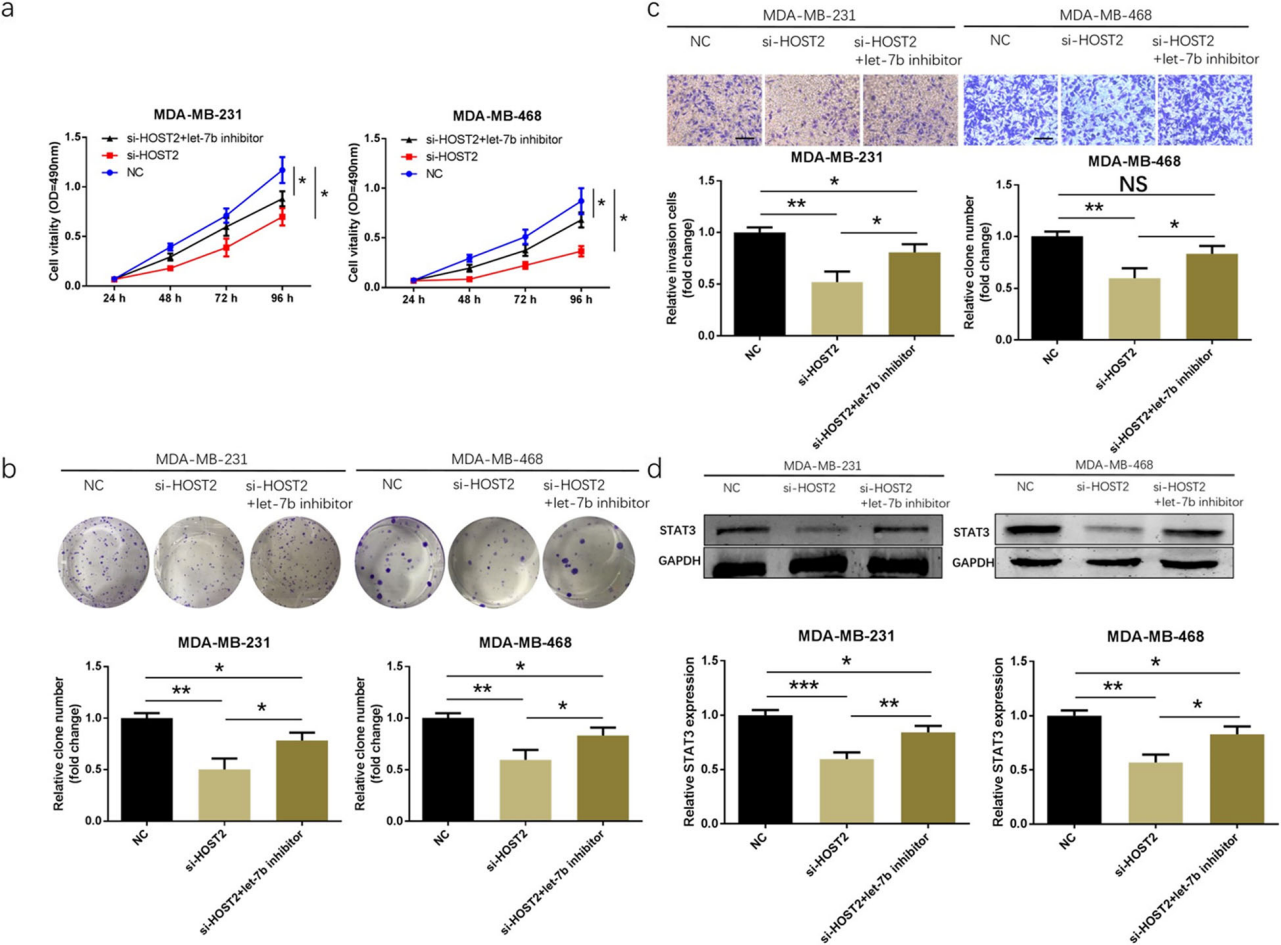
Let-7b knockdown counteracted the tumour suppressing effects of si-HOST2 in TNBC cells. **a-c** Functional rescue experiments were carried out in MDA-MB-231 and MDA-MB-468 cells including MTT assay, colony formation assay, and transwell invasion assay. **d** Western blot analysis showed that knockdown of HOST2 decreased the expression of STAT3 and this effect was reversed by let-7b inhibitor in MDA-MB-231 and MDA-MB-468 cell lines. ^*^P < 0.05, ^**^P < 0.01, ^***^P < 0.001

To discover the molecular mechanism that how let-7b mediates the functions of HOST2 in TNBC cells, let-7b inhibitor as well as si-HOST2 were co-transfected into MDA-MB-231 and MDA-MB-468 cell lines. As shown in Fig. 6a-c, HOST2 knockdown inhibited cell proliferation and invasive potential, while knockdown of let-7b by let-7b inhibitor reversed these tumour suppressing effects. As STAT3 had proved to be a direct target of let-7b, to reveal the indirect regulation relationship between HOST2 and STAT3, TNBC cells were also co-transfected with let-7b inhibitor and si-HOST2, and STAT3 protein level was detected. Western blot analysis suggested that knockdown of HOST2 could downregulate STAT3 expression, and let-7b inhibitor slightly reversed this suppressed effect induced by si-HOST2 (Fig. 6d).

### Knockdown HOST2 inhibited xenograft tumour growth

**Fig. 7:**
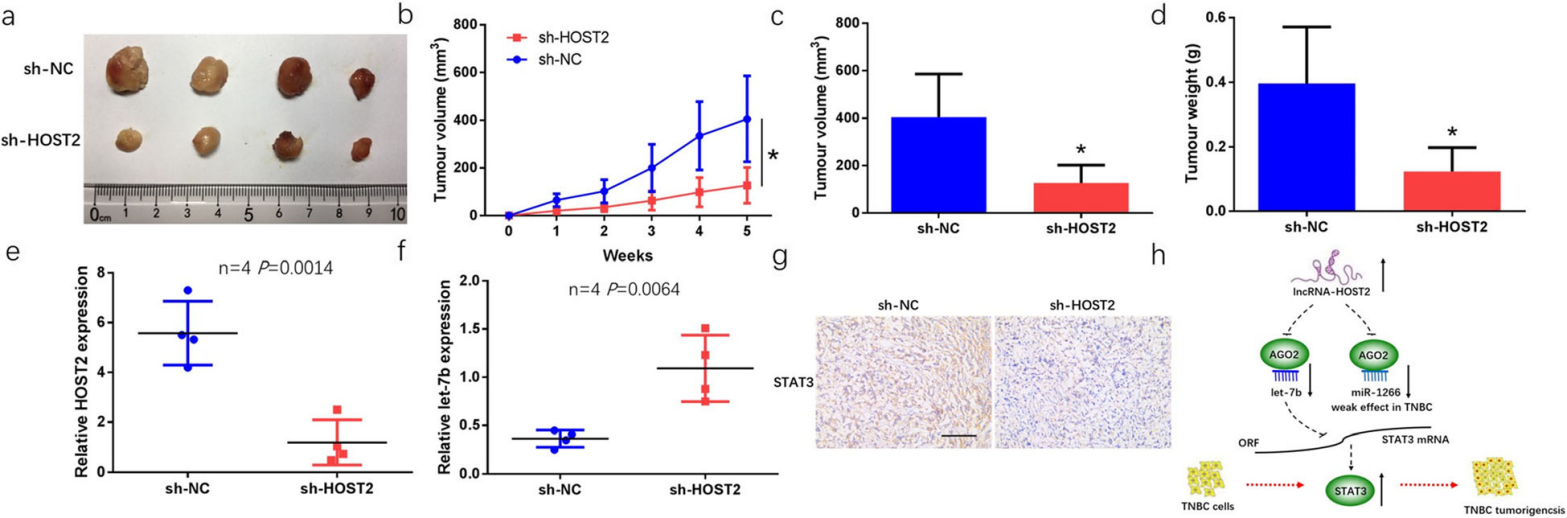
Knockdown of HOST2 inhibited xenograft tumour growth. **a** Representative photographs of xenograft tumour after treatment with sh-HOST2 or sh-NC. **b** Growth curves of tumour proliferation by weeks after treatment with sh-HOST2. **c, d** The average volumes and weight in sh-HOST2 and sh-NC groups after treatment for 5 weeks. **e, f** The expression levels of HOST2 and let-7b in xenograft tumour tissues detected by qRT-PCR analysis. **g** Representative IHC staining of STAT3 stained cells from the indicated tumors (200 × , *n* = 4). **h** HOST2 could sponge let-7b, thereby increasing STAT3 expression and promoting TNBC tumorigenesis. ^*^P < 0.05

In Mice model experiment, MDA-MB-231 cells were transfected with a lentivirus carrying sh-HOST2 or sh-NC precursor sequence. The results showed that the size of the xenograft tumors in sh-HOST2 group was smaller than that in sh-NC group (Fig. 7a). Moreover, the time-dependent analysis showed that the tumour volume was smaller in sh-HOST2 group than sh-NC group (Fig. 7b). Consistently, the average volumes and weight present similar trends as the tumour volume (Fig. 7c, d). After that, the xenograft tumours RNA was extracted, the expression levels of HOST2 and let-7b was detected by qRT-PCR, and the expression of STAT3 was also detected by IHC staining. As shown in Fig. 7e, f, HOST2 expression was decreased and let-7b expression was increased in sh-HOST2 group compared to sh-NC. IHC staining showed that the mean density of STAT3 staining was significantly lower in sh-HOST2 group than sh-NC group (Fig. 7g). In consistent with in vitro results, knockdown of HOST2 markedly decreased the expression of let-7b, but increased STAT3.

## Discussion

Recently, lncRNAs had been discovered as important regulators in BC tumorgenesis [33–35]. HOST2 is reported playing the role of oncogene in diverse carcinomas. In 2015, HOST2 was firstly reported to be over-expressed in human ovarian cancer and functioned as an oncogene in ovarian cancer cells [23]. Liu D reported HOST2 was highly expressed in glioma tissues and its down-regulation could inhibit the growth and invasion of glioma cells [28]. Similar results were reported in hepatocellular carcinoma [24, 25], cervical cancer [26], and gastric cancer [27]. In breast cancer, function of HOST2 in MCF-7 cell (Lumina A type breast cancer cell) was reported [33]. let-7b was identified as the only one miRNA regulated by HOST2 in a few reports [23, 26, 33]. In our study, we found that HOST2 was upregulated in TNBC cell lines and tissue specimens. By investigating the clinicopathological and prognostic significance of HOST2 levels in 40 TNBC tissues, HOST2 was found to be associated with late staging, lymph node metastasis and distant metastasis. Elevated HOST2 was closely correlated with a short survival time in TNBC patients. Based on the above research, we suggested that HOST2 might act as a potential biomarker in TNBC.

Functionally, the potential role of HOST2 in TNBC cells was detected by MTT assay, colony formation assay, EDU assay and transwell invasion assay. These results showed that knockdown of HOST2 markedly suppressed the growth and invasion of TNBC cells in vitro. By mice model experiment, we concluded that knockdown of HOST2 inhibited TNBC tumour growth in vivo. Our functional results were partly similar to previous studies in other types of cancer and other molecular types of breast cancer. Therefore, exploring the detailed mechanism of how HOST2 working in TNBC is meaningful.

For the mechanism part, FISH assay and subcellular fractionation confirmed that HOST2 was enriched in the cytoplasm of TNBC cells. In 2011, the ceRNA hypothesis was proposed, and then widely accepted [36]. Until now, numerous researches had reported that lncRNAs could function as miRNA sponges. For instance, Long non-coding RNA ADPGK-AS1 affects cell proliferation and invasion via miR-542-3p in osteosarcoma [37]. Long non-coding RNA UCA1 can up-regulates PTP1B to enhance cell proliferation through sequestering miR-206 in breast cancer [38]. SNHG8 plays oncogenic roles in the malignancy of esophageal squamous cell carcinoma by sponging miR-411, thus increasing KPNA2 expression [39]. In this study, we identified two miRNAs (let-7b and miR-1266) binding with HOST2 and demonstrated a negative correlation between HOST2 expression and let-7b/miR-1266 in TNBC samples. Knockdown of HOST2 increased the expression level of let-7b/miR-1266 in TNBC cell lines and let-7b/miR-1266 expression was decreased in TNBC tissues. A RIP binding assay proved that HOST2 and let-7b/miR-1266 could bind to Ago2 protein, indicating HOST2 might function as a sponge of one or more miRNAs. It is known that let-7b functions as a tumour suppressor in malignant tumours including breast cancer [40]. However, the role of miR-1266 in TNBC has not been reported. Let-7b and miR-1266 mimics were transfected into MDA-MB-231 and MDA-MB-468 cells. The results suggested that let-7b acted as tumour suppressor in TNBC cells, while effect of miR-1266 in TNBC cells was limited. The potential target of let-7b in TNBC need to be explored. Previous study have shown that let-7b negatively controls IL-8 in breast cancer [41]. Bcl-xL has proved to be another target of let-7b in breast cancer cells [42]. And in esophageal squamous cell carcinoma, let-7 regulates IL-6/STAT3 pathway and is a significant determinant of response to chemotherapy [43]. Consistent with Sugimura K’s study in esophageal squamous cell carcinoma, we here found that let-7b was downregulated in TNBC and had a negative correlation with STAT3 levels. It is well known that the JAK-STAT pathway plays a critical role in cancer. STAT3, a key regulator of JAK-STAT3 signaling pathway, is involved in cell proliferation, survival, migration, invasion, and immunosuppression [44, 45]. It has also been reported that the STAT3 inhibitor, pyrimethamine, displays anti-cancer and immune stimulatory effects in murine models of breast cancer [46]. In addition, antisense experiments were carried on to final support the hypothesis that HOST2 promotes STAT3-mediated cell proliferation and migration via decoying of let-7b in TNBC. Based on these results, we inferred that HOST2 might act as a sponge of let-7b and reduces its activity, thus increasing STAT3 expression and leading to TNBC tumourigenesis (Fig. 7h).

## Conclusions

Our study suggested that the HOST2 is over-expressed in TNBC and regulates STAT3 expression by sponging let-7b. Thus, we identified the HOST2/let-7b/STAT3 axis and its mechanisms and biological effects in TNBC. This axis could serve as a novel therapeutic biomarker for TNBC.

## Supplementary information

**Additional file 1: Figure S1.** The identification of the demarcation point to distinguish high or low expression of HOST2 was obtained by drawing ROC curve. The sensitivity, specificity, and Youden index was calculated. The demarcation point of HOST2 expression locates in the maximum of Youden index.

## Data Availability

The dataset(s) supporting the findings of this study are included within the article.

## Abbreviations

HOST2: Human ovarian cancer specific transcript 2
lncRNA: Long non-coding RNA
TNBC: Triple negative breast cancer
ISH: In situ hybridization
FISH: Fluorescence in situ hybridization
IHC: Immunoprecipitation
MTT: 3-(4,5-dimethyl-2-thiazolyl)-2,5-diphenyl-2-H-tetrazolium bromide
EDU: 5-Ethynyl-2-deoxyuridine
RIP: RNA immunoprecipitation
ER: Estrogen receptor
PR: Progesterone receptor
CeRNAs: Competing endogenous RNAs
ORF: Open reading frame

## References

[1] Cardoso F, Harbeck N, Barrios CH (2017). Research needs in breast cancer. Ann Oncol.

[2] Zhai Q, Li H, Sun L, Yuan Y, Wang X (2019). Identification of differentially expressed genes between triple and non-triple-negative breast cancer using bioinformatics analysis. Breast Cancer.

[3] Mouh FZ, Mzibri ME, Slaoui M, Amrani M (2016). Recent Progress in triple negative breast Cancer research. Asian Pac J Cancer Prev.

[4] Reddy GM, Suresh PK, Pai RR (2017). Clinicopathological features of triple negative breast carcinoma. J Clin Diagn Res.

[5] Thakur V, Kutty RV (2019). Recent advances in nanotheranostics for triple negative breast cancer treatment. J Exp Clin Cancer Res.

[6] Wu Y, Shao A, Wang L (2019). The role of lncRNAs in the distant metastasis of breast Cancer. Front Oncol.

[7] Youness RA, Gad MZ (2019). Long non-coding RNAs: functional regulatory players in breast cancer. Noncoding RNA Res.

[8] Chen X, Sun Y, Cai R (2018). Long noncoding RNA: multiple players in gene expression. BMB Rep.

[9] Tang T, Guo C, Xia T (2019). LncCCAT1 promotes breast Cancer stem cell function through activating WNT/β-catenin signaling. Theranostics.

[10] Liu B, Liu Q, Pan S (2019). The HOTAIR/miR-214/ST6GAL1 crosstalk modulates colorectal cancer procession through mediating sialylated c-met via JAK2/STAT3 cascade. J Exp Clin Cancer Res.

[11] Wang N, Zhang C, Wang W (2019). Long noncoding RNA DANCR regulates proliferation and migration by epigenetically silencing FBP1 in tumorigenesis of cholangiocarcinoma. Cell Death Dis.

[12] Lv P, Qiu X, Gu Y (2019). Long non-coding RNA SNHG6 enhances cell proliferation, migration and invasion by regulating miR-26a-5p/MAPK6 in breast cancer. Biomed Pharmacother.

[13] Panoutsopoulou K, Avgeris M, Scorilas A (2018). miRNA and long non-coding RNA: molecular function and clinical value in breast and ovarian cancers. Expert Rev Mol Diagn.

[14] Pecero ML, Salvador-Bofill J, Molina-Pinelo S (2019). Long non-coding RNAs as monitoring tools and therapeutic targets in breast cancer. Cell Oncol (Dordr).

[15] Bin X, Hongjian Y, Xiping Z (2018). Research progresses in roles of LncRNA and its relationships with breast cancer. Cancer Cell Int.

[16] He L, Hannon GJ (2004). MicroRNAs: small RNAs with a big role in gene regulation. Nat Rev Genet.

[17] Gregory RI, Chendrimada TP, Cooch N (2005). Human RISC couples microRNA biogenesis and posttranscriptional gene silencing. Cell.

[18] Malla RR, Kumari S, Gavara MM (2019). A perspective on the diagnostics, prognostics, and therapeutics of microRNAs of triple-negative breast cancer. Biophys Rev.

[19] Abdollahzadeh R, Daraei A, Mansoori Y (2019). Competing endogenous RNA (ceRNA) cross talk and language in ceRNA regulatory networks: a new look at hallmarks of breast cancer. J Cell Physiol.

[20] Chen C, Zhou L, Wang H (2018). Long noncoding RNA CNALPTC1 promotes cell proliferation and migration of papillary thyroid cancer via sponging miR-30 family. Am J Cancer Res.

[21] Cesana M, Cacchiarelli D, Legnini I (2011). A long noncoding RNA controls muscle differentiation by functioning as a competing endogenous RNA. Cell.

[22] Rangel LB, Sherman-Baust CA, Wernyj RP (2003). Characterization of novel human ovarian cancer-specific transcripts (HOSTs) identified by serial analysis of gene expression. Oncogene.

[23] Gao Y, Meng H, Liu S (2015). LncRNA-HOST2 regulates cell biological behaviors in epithelial ovarian cancer through a mechanism involving microRNA let-7b. Hum Mol Genet.

[24] Liu RT, Cao JL, Yan CQ (2017). Effects of LncRNA-HOST2 on cell proliferation, migration, invasion and apoptosis of human hepatocellular carcinoma cell line SMMC-7721. Biosci Rep.

[25] Wu Y, Yuan T, Wang WW (2018). Long noncoding RNA HOST2 promotes epithelial-Mesenchymal transition, proliferation, invasion and migration of hepatocellular carcinoma cells by activating the JAK2-STAT3 signaling pathway. Cell Physiol Biochem.

[26] Zhang Y, Jia LG, Wang P (2019). The expression and significance of lncRNA HOST2 and microRNA let-7b in HPV-positive cervical cancer tissues and cell lines. Eur Rev Med Pharmacol Sci.

[27] Liu D, Zhang MY, Chu Z, Zhang M (2019). Long non-coding RNA HOST2 enhances proliferation and metastasis in gastric cancer. Neoplasma.

[28] Wang Q, Zhuang ZW, Cheng YM (2019). An in vitro and in vivo study of the role of long non-coding RNA-HOST2 in the proliferation, migration, and invasion of human glioma cells. IUBMB Life.

[29] Noh JH, Kim KM, McClusky WG (2018). Cytoplasmic functions of long noncoding RNAs. Wiley Interdiscip Rev RNA.

[30] Ogunwobi OO, Kumar A (2019). Chemoresistance mediated by ceRNA networks associated with the PVT1 locus. Front Oncol.

[31] Hutvagner G, Simard MJ (2008). Argonaute proteins: key players in RNA silencing. Nat Rev Mol Cell Biol.

[32] Chava S, Reynolds PC, Pathania AS, et al. miR-15a-5p, miR-15b-5p, and miR-16-5p inhibit tumor progression by directly targeting MYCN in neuroblastoma. Mol Oncol. 2019. 10.1002/1878-0261. 10.1002/1878-0261.12588 PMC6944109 31637848

[33] Lu PW, Li L, Wang F, Gu YT (2018). Effects of long non-coding RNA HOST2 on cell migration and invasion by regulating MicroRNA let-7b in breast cancer. J Cell Biochem.

[34] Chang L, Hu Z, Zhou Z, Zhang H (2018). Linc00518 contributes to multidrug resistance through regulating the MiR-199a/MRP1 Axis in breast Cancer. Cell Physiol Biochem.

[35] Li Y, Wang B, Lai H (2017). Long non-coding RNA CRALA is associated with poor response to chemotherapy in primary breast cancer. Thorac Cancer.

[36] Salmena L, Poliseno L, Tay Y (2011). A ceRNA hypothesis: the Rosetta stone of a hidden RNA language?. Cell.

[37] Luo XF, Wu XJ, Wei X (2019). LncRNA ADPGK-AS1 regulated cell proliferation, invasion, migration and apoptosis via targeting miR-542-3p in osteosarcoma. Eur Rev Med Pharmacol Sci.

[38] Li Y, Zeng Q, Qiu J (2019). Long non-coding RNA UCA1 promotes breast cancer by upregulating PTP1B expression via inhibiting miR-206. Cancer Cell Int.

[39] Song H, Song J, Lu L, Li S (2019). SNHG8 is upregulated in esophageal squamous cell carcinoma and directly sponges microRNA-411 to increase oncogenicity by upregulating KPNA2. Onco Targets Ther.

[40] Qin JJ, Yan L, Zhang J, Zhang WD (2019). STAT3 as a potential therapeutic target in triple negative breast cancer: a systematic review. J Exp Clin Cancer Res.

[41] Al-Harbi B, Hendrayani SF, Silva G, Aboussekhra A (2018). Let-7b inhibits cancer-promoting effects of breast cancer-associated fibroblasts through IL-8 repression. Oncotarget.

[42] Wang T, Huang B, Guo R (2015). A let-7b binding site SNP in the 3′-UTR of the Bcl-xL gene enhances resistance to 5-fluorouracil and doxorubicin in breast cancer cells. Oncol Lett.

[43] Sugimura K, Miyata H, Tanaka K (2012). Let-7 expression is a significant determinant of response to chemotherapy through the regulation of IL-6/STAT3 pathway in esophageal squamous cell carcinoma. Clin Cancer Res.

[44] Yu H, Lee H, Herrmann A, Buettner R, Jove R (2014). Revisiting STAT3 signalling in cancer: new and unexpected biological functions. Nat Rev Cancer.

[45] Banerjee K, Resat H (2016). Constitutive activation of STAT3 in breast cancer cells: a review. Int J Cancer.

[46] Khan MW, Saadalla A, Ewida AH (2018). The STAT3 inhibitor pyrimethamine displays anti-cancer and immune stimulatory effects in murine models of breast cancer. Cancer Immunol Immunother.

