# Supplementary figures and images for: Long noncoding RNA HOST2, working as a competitive endogenous RNA, promotes STAT3-mediated cell proliferation and migration via decoying of let-7b in triple-negative breast cancer
Source: J Exp Clin Cancer Res. 2020 Apr 5;39:58. doi: 10.1186/s13046-020-01561-7 (PMC7132993; doi:10.1186/s13046-020-01561-7)

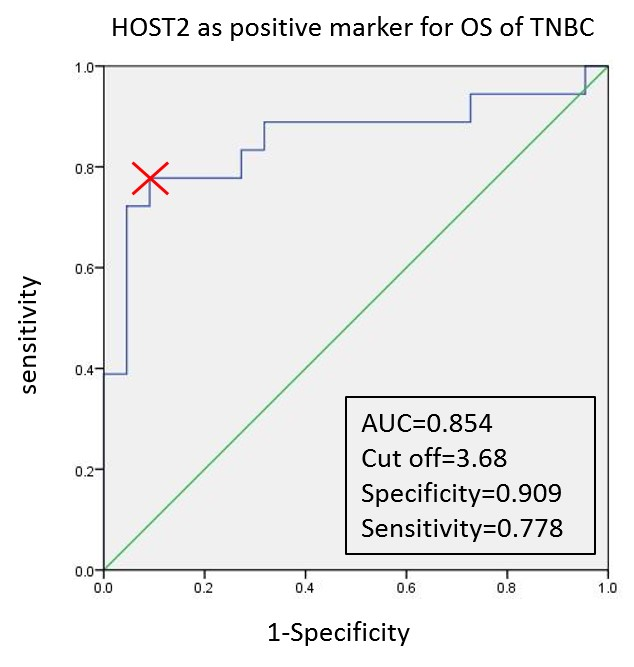

Supplement: Supplementary file 1 — Additional file 1: Figure S1. The identification of the demarcation point to distinguish high or low expression of HOST2 was obtained by drawing ROC curve. The sensitivity, specificity, and Youden index was calculated. The demarcation point of HOST2 expression locates in the maximum of Youden index. [file 13046_2020_1561_MOESM1_ESM.tif]
